# Molecular Identification of *Leishmania* Species in *Phlebotomus alexandri* (Diptera: Psychodidae) in Western Iran

**DOI:** 10.18502/jad.v14i1.2699

**Published:** 2020-03-31

**Authors:** Abdollah Naghian, Mohammad Ali Oshaghi, Vahideh Moein-Vaziri, Yavar Rassi, Mohammad Mehdi Sedaghat, Ehsan Mostafavi, Arshad Veysi, Hassan Soleimani, Hossein Dehghan, Alireza Zahraei-Ramazani, Hossein Mirhendi, Mohammad Hassan Amini, Mohammad Reza Yaghoobi-Ershadi, Amir Ahmad Akhavan

**Affiliations:** 1 Department of Medical Entomology and Vector Control, School of Public Health, Tehran University of Medical Sciences, Tehran, Iran; 2 Department of Medical Entomology and Vector Control, School of Public Health, Urmyia University of Medical Sciences, Urmyia, Iran; 3 Department of Medical Parasitology and Mycology, School of Medicine, Shahid Beheshti University of Medical Sciences, Tehran, Iran; 4 Department of Epidemiology, Pasteur Institute of Iran, Tehran, Iran; 5 Zoonoses Research Center, Research Institute for Health Development, Kurdistan University of Medical Sciences, Sanandaj, Iran; 6 Yazd Health Research Station, National Institute of Health Research, Yazd, Iran; 7 Department of Public Health, School of Public Health, Jiroft University of Medical Sciences, Jiroft, Iran; 8 Department of Medical Parasitology and Mycology, School of Medicine, Isfahan University of Medical Sciences, Esfahan, Iran

**Keywords:** Leishmaniasis, *Phlebotomus alexandri*, *Leishmania*, Iran

## Abstract

**Background:**

Visceral and cutaneous leishmaniasis are common in some areas of Iran and consider as health problems. *Phlebotomus alexandri* has been incriminated as a suspected vector for the both form of leishmaniasis.

**Methods:**

This study was carried out in 4 western provinces of Iran. Sand flies were collected using sticky traps and light traps from indoor and outdoor resting places. Nested PCR was employed to detect *Leishmania* parasites among collected sand flies.

**Results:**

Seven hundred and twenty two *P. alexandri* females were collected and pooled in 179 batches. Results of nested PCR showed, out of 9 samples from East Azerbaijan Province, only one sample was infected by *Leishmania infantum*. Of 34 individual and pooled samples from Kermanshah Province, only one pooled sample was infected with *Leishmania major* and among 30 individual and pooled samples in Fars Province, five specimens were infected by *L. major*, *L. infantum*, *Leishmania donovani* and *Leishmania tropica.* Furthermore*,* out of 108 individual and pooled samples from Khuzestan Province, 10 samples showed infection with *L. major* and *L. infantum*.

**Conclusion:**

The results of this study showed that *P. alexandri* is more active in hot zones than in moderate zones and this species may be considered as a permissive species.

## Introduction

Leishmaniasis is a parasitic infectious disease that is transmitted by the bite of infected phlebotominae sandflies, and it is grouped in the seventeen neglected tropical diseases (NTD) (1). Which has been recorded from 98 countries, (2). There are three main forms of disease in the world including visceral leishmaniasis (VL), cutaneous leishmaniasis (CL) and mucocutaneous leishmaniasis (MCL) (3). So far two types of the disease including CL and VL, has been reported in Iran (4, 5). Cutaneous leishmaniasis is the most common form of the disease in the world as well as in Iran (5, 6). Although the new VL foci have increased remarkably, zoonotic visceral leishmaniasis (ZVL) incidence has decreased recently in Iran (Ministry of Health and Medical Education (MOHME), 2015, unpublished data). There are about 1000 sand fly species and subspecies reported in the world (7), of these only about 93 species transmit 20 *Leishmania* species to humans (2). Till now, 29 species and 2 subspecies of *Phlebotomus* and 19 species of *Sergentomyia* sand flies were reported in Iran (8). Six sand fly species including *Phlebotomus salehi*, *P. mongolensis*, *P. alexandri*, *P. andrejevi*, *P. caucasicus* and *P. ansarii* known as suspected vectors and *P. papatasi* known as the main proven vector of zoonotic cutaneous leishmaniasis (ZCL) due to *L. major* in Iran (4). Moreover, anthroponotic cutaneous leishmaniasis (ACL) due to *L. tropica* occurs in 14 foci of 7 provinces in Iran. Four species of the subgenus *Larrussious* and *P. alexandri* of the subgenus *Paraphlebotomus* were incriminated as probable vectors of ZVL due to *L. infantum* in seven new and old foci of the country (4, 5). *Phlebotomus alexandri* reported as a proven vector for ZVL (2) and anthroponotic visceral leishmaniasis (AVL) in china (9). Furthermore, it is incriminated as the probable vector of AVL and ZVL in Iraq, Oman, Mongolia, Turkey and China (2, 10). *Leishmania* infection rate for *P. alexandri* reported as 1.74% in Khuzestan endemic regions (11). It was also found infected by *L. infantum* (12) and *L. major* (13) using molecular methods in Fars Province in south west and Sarakhs in north eastern of Iran respectively. Furthermore, *P. alexandri* was found infected in Turkemanistan (14) and it is suspected to transmission of *L. killicki* in Tunisia (15, 16). Considering the studies have been conducted so far in Iran and other countries, it seems *P. alexandri* is a permissive species, however more studies are needed to clarify the role of *P. alexandri* in different *Leishmania* parasite transmission. The current study was designed and conducted in four western endemic and non-endemic provinces of Iran.

## Materials and Methods

### Study area

This study was carried out in 4 western and south western provinces of Iran including Eastern Azerbaijan, Fars, Kermanshah and Khuzestan from 2011 to 2012. In Eastern Azerbaijan Province (36°, 45’ to 39°, 26’ N and 45° 5’ to 48° 21’ E) in Northwest of Iran, maximum and minimum temperature were +45 °C in Julfa and −25 °C in Bostanabad respectively and the rainfall varied between 196 to 563 mm. In Kermanshah Province (33°, 41’ to 35°, 17’ N and 45° 24’ to 48° 6’ E) in the West of Iran, maximum and minimum temperature were +50.4 °C in Summer and −17.4 °C in Sonqur respectively and the rainfall varied between 273 to 573 mm. In Fars Province (27°, 3’ to 31°, 40’ N and 50° 36’ to 55° 35’ E) in South West of Iran, maximum and minimum of temperature were +49.8 °C in Lamard and −14.6 °C in Safashahr respectively and the rainfall varied between 83 to 1007 mm. In Khuzestan Province (29°, 53’ to 33°, 0’ N and 47° 40’ to 50° 33’E) in south west of Iran, maximum and minimum of recorded temperature in this province were +52.6 °C in Shushtar and −5.6 °C in Dehdaz respectively and the rainfall varied between 73 to 702mm (17).

### Sand fly collection and identification

**Fig. 1.**
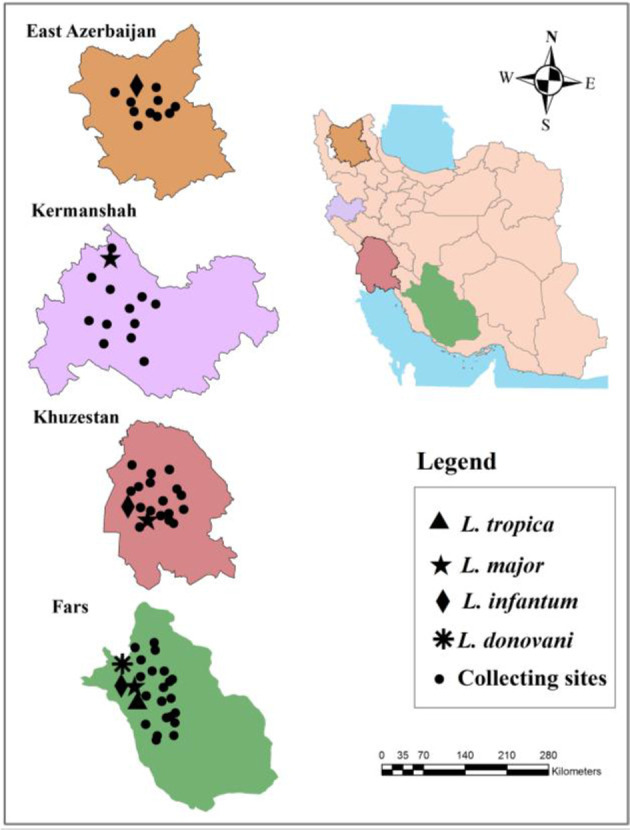
The map of *Leishmania infection* of *Phlebotomus alexandri* in the studied areas, Iran, 2011 and 2012

Sand flies were collected using light traps and sticky traps during active seasons of sand flies in 127 places and 599 places in 2011 (Fig. 1). Collected sand flies were conserved in 96% alcohol, kept in 4 °C refrigerator. The head and the last two segments of sand flies were dissected on the slide in sterile condition and mounted in Puris media. The rest of the bodies including abdomen and thorax were transferred into a sterile 1.5ml micro tube and were kept in −20 °C until use. The specimens were identified using valid morphological keys (18, 19).

### Molecular experiments

Genomic DNA was extracted and purified using a Geneall kit (Exgene™ Tissue SV mini) according to the protocol of the kit.

### PCR conditions

Polymerase Chain Reaction amplification has done in an Applied Biosystems thermocycler. The first step of Nested-PCR contained 0.6μM of each forward (AAACTCCTCTCT GGTG-CTTGC; Leish out F) and reverse (AAA CAAAGGTTGTCGGGGG; Leish out R) external primers (20), 12.5μl Taq DNA polymerase, 2X Master Mix Red (Amplicon, Denmark) and sterile distilled water to a final volume of 25 μl. First denaturation step at 95 °C for 5min was continued by 30 cycles of: denaturation at 94 °C for 30s, annealing at 60 °C for 45s and extension at 72 °C for 1min, with a final extension step of 72 °C for 5min. The second step of Nested-PCR was performed in a final volume of 20μl containing 1μl of a 1/10 dilution in distilled water of the first-round PCR product as template, 0.3μM of each forward (AATTCAACTTCGCGTT GGCC; Leish in F) and reverse (CCTCTC TTTTTTCTC-TGTGC; Leish in R) (20), 10μl of Taq DNA polymerase and 2X Master mix Red. The second round PCR was cycled under the following conditions: 95 °C for 2min, 25 cycles of 94 °C for 15s, 62 °C for 30s, 72 °C for 45s followed by 72 °C for 5min. PCR products were electrophoresed on 1.5% (w/v) agarose gel in TBE buffer (0.09mM Tris, 0.09mM boric acid and 20mM EDTA, pH 8.3), visualized with safe stain (0.5μg/ml) and photographed.

Reference strains of *L. infantum*, *L. major* and *L. tropica* were used as positive controls and distilled water was used as negative control accordingly. The PCR product of the negative control of the first step of PCR was used as the negative control in the second step and the PCR product of the positive control of the first step PCR was used as positive control in the second step as well. To avoid cross-contamination, necessary precautions were taken. The products of nested PCR were sent to corresponding company for nucleotide sequencing.

## Results

Sand flies were collected using 207 light traps and 7633 sticky traps during active seasons of sand flies in the study areas. Totally, 722 female *P. alexandri* were collected in 4 studied provinces including 9 from East Azerbaijan, 114 from Fars, 90 from Kermanshah, and 509 specimens from Khuzestan. Collected sand flies were pooled in 179 batches according to five parameters including: locality (counties of provinces), places of catch (indoor or outdoor), type of trap (light trap or sticky trap), topography (mountains, foothills or plains) and physiological status (unfed, blood-fed, semigravid and gravid) of the sand flies. The location where *P. alexandri* sand flies were caught and their *Leishmania* infection are shown in (Fig. 1).

**Fig. 2.**
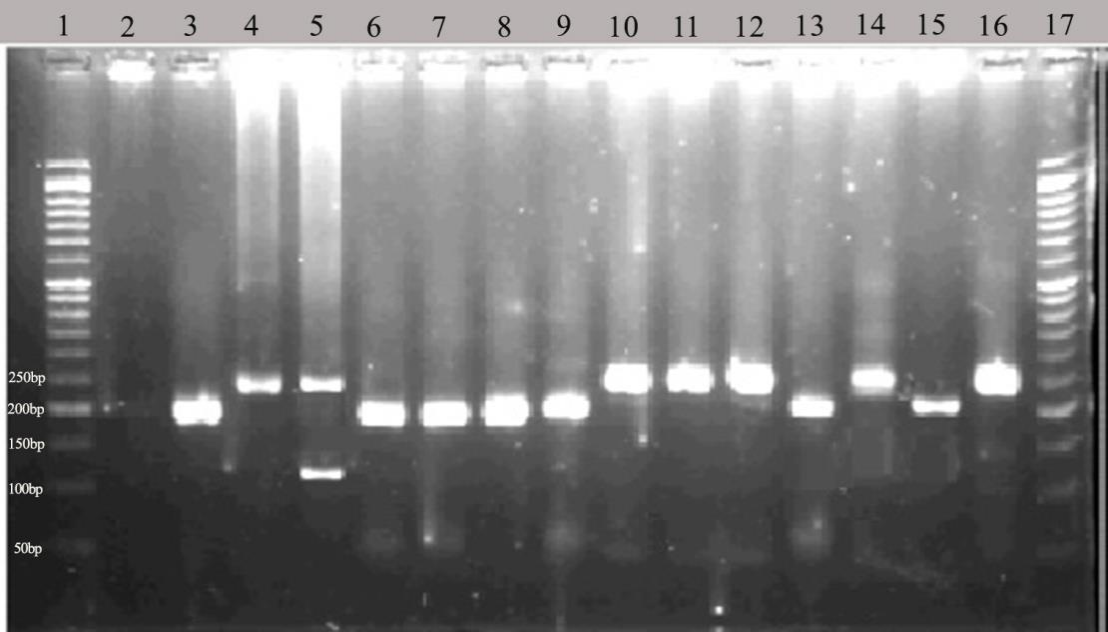
The 1.5% agarose gel electrophoresis of nested PCR products in Khuzestan and Kermanshah Provinces samples. Lanes 1 and 17 are 50bp ladder; lane 2- negative control; lane 3- *Leishmania infantum* positive control; lane 4- *Leishmania major* positive control; lane 5- *Leishmania tropica* positive control; lane 6–15- samples of Khuzestan Province; lane 16 sample from Kermanshah Province

In Kermanshah Province, of 34 pooled samples, only one was infected by *L. major* (Fig. 2). This sample was contained 13 unfed female *P. alexandri* sand flies, which collected from 4 villages (Kalashi Lulem, Kalashi Bakhan, Mezran and Melah Rash) of Javanrud County (Table 1).

**Fig. 3.**
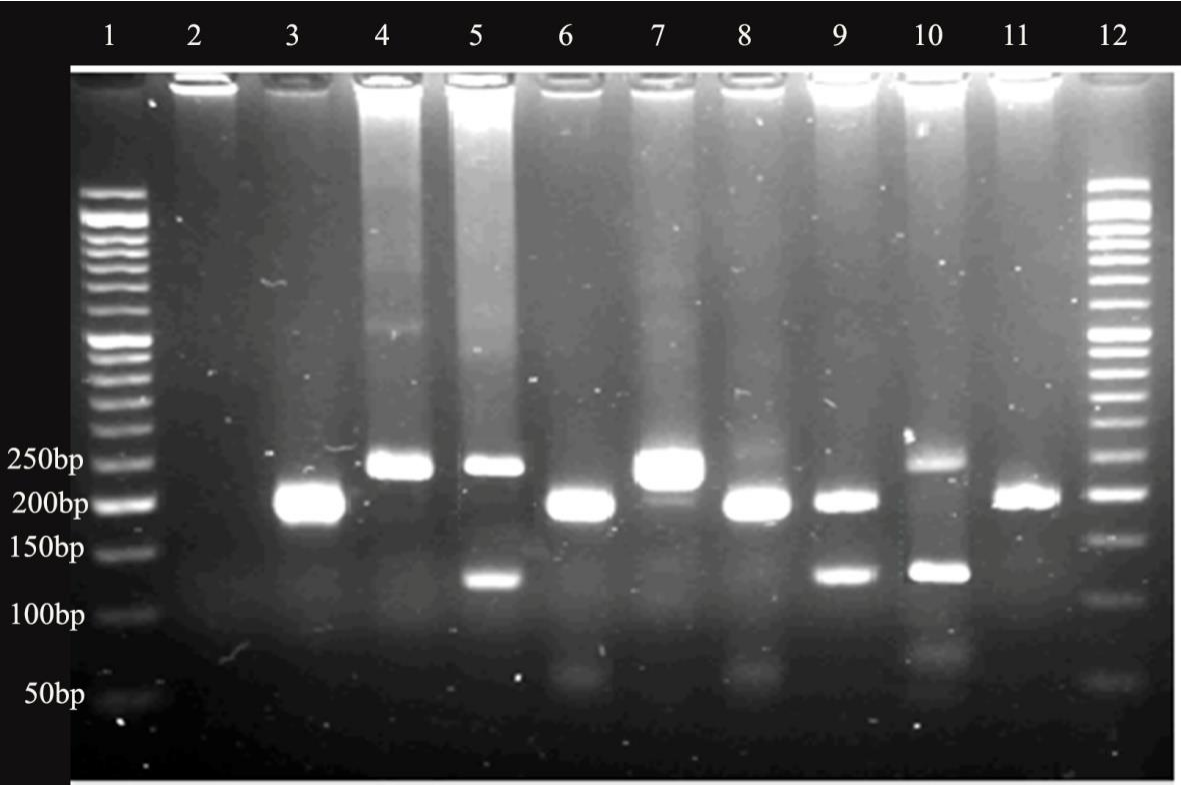
The gel electrophorus image of nested PCR results in Fars and Eastern Azerbaijan provinces’ samples. Lene 1 and 12 are 50bp ladder; Lane 2 is negative control; Lane 3, 4 and 5 are positive control of *Leishmania infantum*, *L. major* and *Leishmania tropica*, respectively; lane 7–11 samples of sand flies from studied areas

**Table 1.** The geographical and epidemiological parameters of positive pooled samples in the studied regions

**Geographical status**	**Province**	**Place**	**Samples cods**	***Leishmania* species**	**No. sand flies in sample**
**Plain**	Khuzestan	Indoor	108	*L. infantum*	1
		Indoor	98.3	*L. infantum*	23
		Outdoor	119	*L. infantum*	2
		Indoor	97.3	*L. major*	16
		Indoor	89.2	*L. major*	18
		Indoor	89.1	*L. major*	4
		Outdoor	152	*L. infantum*	1
		Outdoor	116	*L. major*	1
**Foothills**	Fars	Outdoor	24.2	*L. major*	14
		Indoor	3	*L. infantum*	3
		Outdoor	19	*L. major* and *L. tropica*	1
		Indoor	2m	*L. infantum* and *L. tropica*	1
**Mountain**	Khuzestan	Indoor	122	*L. infantum*	1
	Fars	Outdoor	28	*L. donovani*	1
	Kermanshah	Outdoor	41	*L. major*	13
	East Azerbaijan	Outdoor	65.1	*L. infantum*	1

In Eastern Azerbaijan, collected *P. alexandri* sand flies were pooled in 9 samples. Among these samples only one was infected by *L. infantum* (Fig. 1, Table 1). The infected sample was contained 1 unfed female sand fly that was collected in a mountainous cave near Ghyzil gaya in Ahar County.

In Fars Province, collected *P. alexandri* sand flies were pooled in 31 batches. Of these 5 samples were infected by *leishmania* parasites (Fig. 1, Table 1). Two samples were infected by *L. infantum.* One of the infected sample by *L. infantum* was contained 3 blood-fed *P. alexandri* female which were collected in 2 villages (Shorab, ChamGol, and PirehSorkh) of Mamasani County. One of the infected sample was infected by *L. donovani* contained an unfed *P. alexandri* female that was collected in a cave located in Nur Abad City. The other one showed a mixed infection of *L. infantum* and *L. tropica*, contained an unfed *P. alexandri* female which was collected in Pireh Sorkh Village of Mamasani.

Two samples were found infected by *L. major*, one of them was collected in Khumeh Zar Village of Mamasani County that was a pooled of 14 unfed female sand flies and another was a mixed infection of *L. major* and *L. tropica* contained an unfed female that was collected in Shir Espari Village of Mamasani County.

In Khuzestan Province, all collected *P. alexandri* female sand flies were pooled in 108 samples, of these 10 samples were infected by *Leishmania* parasites and all were unfed. Results showed, in Seyyed Taher Village that located in plain area in north of Ahvaz County, three samples were infected by *L. major*, and three samples were infected by *L. infantum* (Fig. 1, Table 1). Furthermore, another sample was infected with *L. infantum* in Sar Dasht (Kohe Gandom) village in mountainous area in north of Dezful County (Fig. 1). Apart from these, a sample contained one *P. alexandri* sand fly had infection of *L. infantum* was collected in Dehenow Bagher Village located in plain area in south of Dezful County, (Fig. 1, Table 1). Moreover, a sample contained a sand fly that was infected with *L. major* collected in Magtu Village in the south of Ahvaz County, (Fig. 1, Table 1). The geographical and epidemiological parameters of pooled samples are shown in Table 1.

## Discussion

The result of this study showed that, *P. alexandri* harbored a couple of leishmania parasites in the endemic and non-endemic areas. Sand flies infection by *L. major* from Javanrud County villages in Kermanshah Province showed, despite there is no CL cases in these areas, it is postulated that, there is an enzootic foci in this region which can pose a risk to extend ZCL to people in the future. This is the first report of *P. alexandri* sand fly infection due to *L. major* in Javnrud County in Kermanshah Province. This region is located near Qasr-e Shirin and Sare Pole Zahab Counties, where the previous studies reported ZCL cases among endogenous people (21). In East Azerbaijan, the study regions are located near Meshkin Shahr City in the Ardebil Province where has been known as the first main endemic foci of ZVL in Iran (4). The infection of *P. alexandri* due to *L. infantum* has been reported in Fars Province as well (12)*.* Recently, *P. tobbi* was found infected with *L. infantum* in East Azerbaijan (22). In northwest of Iran, *P. kandelakii* (23) and *P. perfiliewei* (24) were reported infected by *L. infantum* as well. Here, *P. alexandri* has been introduced as the fourth suspected ZVL vector in the north west of Iran. Since the infected *P. alexandri* with *L. infantum* was collected in a fox nest in the border of Karamlu and Khalifan Villages of Ahar County, it implies that foxes can play a role in enzootic cycle of parasite in this region. The results of our studies in Fars Province showed that, the infection of *P. alexandri* with *Leishmania* parasites is higher comparing to other provinces. This province known as ACL and ZVL foci in the country (4). According to recent report of Ministry of Health, the occurrence of CL in this province is very high. *Leishmania major* infection in *P. alexandri* is reported for the first time in Mamasani County in Fars Province, although *L. infantum* infection in *P. alexandri* has been reported in this area previously (12). Since the occurring mixed infection in *P. alexandri*, this species might play role as a permissive vector which supports multiple *Leishmania* parasite inside the digestive tract. Additionally, it is the first report of *P. alexandri* infection with *L. donovani* in Nur Abad City as well. In the current study *P. alexandri* found infected with *L. infantum* in Chamgol and Pireh Sorkh Villages of Mamasani County for the first time in Fars Province. In Khuzestan Province, *P. alexanderi* was found infected with 2 *Leishmania* parasites including, *L. major* and *L. infantum*. On the other hand, in East Azerbaijan, *P. alexandri* was found infected with *L. infantum* in a fox nest as well. In contrast to the results of previous studies and also current study in other regions, *P. alexandri* mostly collected in the plain areas in Khuzestan Province. According to the MOHME internal reports in 2015, Fars and Khuzestan Provinces had the first and second incidences of CL in Iran respectively, but Kermanshah Province had the ninth incidence at the same time. Also these reports state that Fars and East Azerbaijan Provinces had the first and second incidence of ZVL in Iran respectively, but Khuzestan had just 1 case in the year 2015. Microscopic Leishmania infection of *P. alexandri* in Shush and Ahvaz Counties was reported 4 decades ago, but was not detected by molecular techniques and did not show any successful inoculation in sensitive animals (25). In agreement with previous work, our study introduced *P. alexandri* as a probable vector of ZCL and ZVL in Iran. In the current study the majority of infected *P. alexandri* sand flies was unfed and it reflects that, they are capable to transmit the parasite to the new host through next blood feeding. Furthermore, the finding highlights that *P. alexandri* mostly occurred in hot regions (Khuzestan) rather than cold regions (other studied provinces). This is the first report of *L. infantum*, *L. donovanoi* and *L. major* infections in *P. alexandri* in Khuzestan Province, and is the first report of infection to *L. tropica* by *P. alexandri* using molecular technique in the world. In Khuzestan Province, the occurrences of CL is common according to the Iranian MOHME reports, but only 1 case was reported in this province for ZVL in 2015 (26). On the other hand according to WHO and some other studies, in eastern provinces of Iraq that are located on the border of Khuzestan (Missan and Basrah) and other provinces on the border of Ilam and other western provinces (Diala and Wasit), the ZVL is endemic (27–29). The 90% of ZVL cases in Iraq occur among less than 5 years old children. The number of reported cases were 3900 in Iraq in 1992 and increased to 1050 cases in 2012 (28).

## Conclusion

The results of this study showed, *P. alexandri* could harbor infection of 4 *Leishmania* species (*L. major*, *L. tropica*, *L. donovani* and *L. infantum*) so that, it supports the hypothesis of permissiveness of this species. Furthermore, the reporting of natural infection of *P. alexanderi* in some presumably free areas of ZCL, e.g. in Kermanshah, revealed the cycling of parasite among its host and sand flies vector at least in enzootic cycle.

## References

[1] WHO (2015) Investing to overcome the global impact of neglected tropical diseases: third WHO report on neglected tropical diseases. World Health Organization, Geneva, p. 211.

[2] WHO (2010) Control of the leishmaniasis. WHO technical report sereis 949. Report of the meeting of the WHO expert committee on the control of leishmaniasis, Geneva.

[3] Desjeux P (2004) Leishmaniasis: current situation and new perspectives. Comp Immunol Microbiol Infect Dis. 27(5): 305–318. 15225981 10.1016/j.cimid.2004.03.004

[4] Yaghoobi-Ershadi MR (2012) Phlebotomine Sand Flies (Diptera: Psychodidae) in Iran and their Role on Leishmania Transmission. J Arthropod Borne Dis. 6(1): 1–17. 23293774 PMC3528173

[5] Yaghoobi-Ershadi MR (2016) Control of phlebotomine sand flies in Iran: a review article. J Arthropod Borne Dis. 10(4): 429–444. 28032095 PMC5186733

[6] Alvar J Vélez ID Bern C Herrero M Desjeux P Cano J Jannin J den Boer M Team WLC (2012) Leishmaniasis worldwide and global estimates of its incidence. PloS One. 7(5): e35671. 22693548 10.1371/journal.pone.0035671 PMC3365071

[7] Bates PA Depaquit J Galati EA Kamhawi S Maroli M McDowell MA Picado A Ready PD Salomón OD Shaw JJ (2015) Recent advances in phlebotomine sand fly research related to leishmaniasis control. Parasit Vectors. 8(1): 131. 25885217 10.1186/s13071-015-0712-x PMC4352286

[8] Karimi A Hanafi-Bojd AA Yaghoobi-Ershadi MR Akhavan AA Ghezelbash Z (2014) Spatial and temporal distributions of phlebotomine sand flies (Diptera: Psychodidae), vectors of leishmaniasis, in Iran. Acta Trop. 132: 131–139. 24462940 10.1016/j.actatropica.2014.01.004

[9] Guan LR Xu YX Li B-S Dong J (1986) The role of *Phlebotomus alexandri Sinton*, 1928 in the transmission of kala-azar.Bull World Health Organ. 64 (1):107–112. 3488133 PMC2490915

[10] Killick-Kendrick R (1990) Phlebotomine vectors of the leishmaniases: a review. Med Vet Entomol. 4(1): 1–24. 2132963 10.1111/j.1365-2915.1990.tb00255.x

[11] Javadian E Mesghali A Nadim A (1977) Natural leptomonad infection of sand flies with its first occurrence in *P. alexandri* in Khuzistan Province, Iran. Ecologie de Leishmaniasis. Coll Int CNRS. 239: 203–205

[12] Azizi K Rassi Y Javadian E Motazedian M Rafizadeh S Yaghoobi-Ershadi MR Mohebali M (2006) *Phlebotomus* (*Paraphlebotomus*) *alexandri*: a probable vector of *Leishmania infantum* in Iran. Ann Trop Med Parasitol. 100(1): 63–68. 16417715 10.1179/136485906X78454

[13] Bakhshi H Oshaghi M Abai M Rassi Y Akhavan A Sheikh Z Mohtarami F Saeidi Z Mirzajani H Anjomruz M (2013) Molecular detection of *Leishmania* infection in sand flies in border line of Iran-Turkmenistan: Restricted and permissive vectors Exp Parasitol. 135(2): 382–387. 23933280 10.1016/j.exppara.2013.07.020

[14] Petriščeva P (1971) The natural focality of leishmaniasis in the USSR. Bull World Health Organ. 44(4): 567–76. 5316258 PMC2427832

[15] Colacicco-Mayhugh MG (2009) Biology and ecology of sand flies (diptera: psychodidae) in the middle east, with special emphasis on *Phlebotomus papatasi* and *Phlebotomus alexandri*. (Ph.D. Thesis) Department of Preventive Medicine and Biometrics Uniformed Services University, of the health sciences F. Edward Herbert School of Medicine.

[16] Ghrab J Rhim A Bach-Hamba D Chahed M Aoun K Nouira S Bouratbine A (2006) Phlebotominae (Diptera: Psychodidae) of human leishmaniosis sites in Tunisia. Parasite. 13(1): 23–33. 16605064 10.1051/parasite/2006131023

[17] Iran Ministry of Roads and Urban Development, National Meteorological Organization Available at: http://www.irimo.ir/far/wd/2703

[18] Seyedi-Rashti M Nadim A (1992) The genus Phlebotomus (Diptera: Psychodidae: Plebotominae) of the countries of the Eastern Mediterrenean Region. Iran J Public Health. 21(1–4): 11–50.

[19] Theodor O Mesghali A (1964) On the phlebotominae of Iran. J Med Entomol. 1: 285–300. 14222870 10.1093/jmedent/1.3.285

[20] Akhavan AA Mirhendi H Khamesipour A Alimohammadian MH Rassi Y Bates P Kamhawi S Valenzuela JG Arandian MH Abdoli H (2010) *Leishmania* species: Detection and identification by nested PCR assay from skin samples of rodent reservoirs. Exp parasitol. 126(4): 552–556. 20566364 10.1016/j.exppara.2010.06.003 PMC2939322

[21] Hamzavi Y Sobhi S Rezaei M (2009) Epidemiological factors of cutaneous leishmaniasis in the patients referred to health centers in Kermanshah Province. Behbood Journal. 13(2): 151–161.

[22] Rassi Y Sanei-Dehkordi Ar Oshaghi MA Abai MR Mohtarami F Enayati A Zarei Z Javadian E (2012) First report on natural infection of the *Phlebotomus tobbi* by *Leishmania infantum* in northwestern Iran. Exp parasitol. 131(3): 344–349. 22609304 10.1016/j.exppara.2012.04.020

[23] Rassi Y Javadian E Nadim A Zahraii A Vatandoost H Motazedian H Azizi K Mohebali M (2005) Phlebotpmus (Larroussius) kandelakii the Principal and Proven Vector of Visceral Leishmaniasis in North West of Iran. Pak J Biol Sci. 8: 1802–1806.

[24] Sanei-Dehkordi AR Rassi Y Oshaghi M Abai M Rafizadeh S Yaghoobi-Ershadi M Mohebali M Zarei Z Mohtarami F Jafarzadeh B Javadian E (2011) Molecular detection of *Leishmania infantum* in naturally infected *Phlebotomus perfiliewi* transcaucasicus in Bilesavar District, northwestern Iran. Iran J Arthropod Borne Dis. 5(1): 20–27. 22808407 PMC3385567

[25] Javadian E Mesghali A (1974) Studies on cutaneous leishmaniasis in Khuzestan, Iran. Part I. The leptomonad infection of sandflies. Bull Soc Pathol Exot Filiales. 67(5): 513–516. 4480471

[26] Iran Ministry of Health and Medical Education (2016) Infectious Disease Management, Annual Meeting of the Zoonosis Group in Sarein, Ardabil.

[27] WHO (2003) Communicable Disease Working Group on Emergencies, HQ Division of Communicable Disease Control, EMRO, WHO OFFICE, Baghdad. WHO Office, Baghdad Communicable Disease Toolkit, IRAQ CRISIS. WHO 2003: 39–44.

[28] Salam N Al-Shaqha WM Azzi A (2014) Leishmaniasis in the Middle East: incidence and epidemiology. PLoS Negl Trop Dis. 8 (10): e3208. 25275483 10.1371/journal.pntd.0003208 PMC4183486

[29] Rahi AA Ali MA Valian HK Mohebali M Khamesipour A (2013) Seroepidemiological studies of visceral leishmaniasis in Iraq. Sch J App Med Sci. 1(6): 985–989.

